# Multiple Strategies for Translesion Synthesis in Bacteria

**DOI:** 10.3390/cells1040799

**Published:** 2012-10-15

**Authors:** Paul J. Ippoliti, Nicholas A. DeLateur, Kathryn M. Jones, Penny J. Beuning

**Affiliations:** 1 Department of Chemistry and Chemical Biology, Northeastern University, Boston, MA 02115, USA; Email: ippoliti.paul@gmail.com (P.J.I.); nicholas.delateur@gmail.com (N.A.D.); 2 Department of Biological Science, Florida State University, Tallahassee, FL 32306, USA; Email: kmjones@bio.fsu.edu; 3 Center for Interdisciplinary Research on Complex Systems, Northeastern University, Boston, MA 02115, USA

**Keywords:** DNA damage, mutagenesis, SOS response, DNA pol IV (DinB), DNA pol V (UmuD'_2_C), *dnaE*, *dnaE2*, *imuA*, *imuB*, *imuC*

## Abstract

Damage to DNA is common and can arise from numerous environmental and endogenous sources. In response to ubiquitous DNA damage, Y-family DNA polymerases are induced by the SOS response and are capable of bypassing DNA lesions. In *Escherichia coli*, these Y-family polymerases are DinB and UmuC, whose activities are modulated by their interaction with the polymerase manager protein UmuD. Many, but not all, bacteria utilize DinB and UmuC homologs. Recently, a C-family polymerase named ImuC, which is similar in primary structure to the replicative DNA polymerase DnaE, was found to be able to copy damaged DNA and either carry out or suppress mutagenesis. ImuC is often found with proteins ImuA and ImuB, the latter of which is similar to Y‑family polymerases, but seems to lack the catalytic residues necessary for polymerase activity. This *imuAimuBimuC* mutagenesis cassette represents a widespread alternative strategy for translesion synthesis and mutagenesis in bacteria. Bacterial Y‑family and ImuC DNA polymerases contribute to replication past DNA damage and the acquisition of antibiotic resistance.

## 1. Introduction

Various endogenous and exogenous agents can cause damage to DNA, creating lesions and leading to mutagenesis [1]. Replicative DNA polymerases, enzymes that catalyze DNA replication, are incapable of replicating damaged DNA [1], although this inability is not absolute [2,3,4,5]. Therefore, cells across all domains of life are equipped with specialized DNA polymerases that have the ability to replicate damaged DNA in a process known as translesion synthesis (TLS), which was first proposed over 30 years ago [6,7,8,9]. This family of specialized DNA polymerases is known as the Y family of DNA polymerases, after the X family, because Y-family DNA polymerases play a greater role in DNA damage tolerance than in chromosomal replication [8,10]. Despite this specialized function to bypass damaged DNA, Y-family polymerases may cause mutations, because they are unable to replicate undamaged DNA with the same high fidelity as typical replicative polymerases [6,7]. These two functions have important implications for human health, as the mutagenic functions of Y family DNA polymerases contribute to antibiotic resistance, whereas the damage bypass functions can both prevent damage-induced mutations that can lead to cancer as well as decrease the effectiveness of DNA‑damaging cancer chemotherapy drugs by allowing cells to tolerate such damage [11]. This review will focus on the two *E. coli* Y-family polymerases as well as Y-family and other specialized DNA polymerases found in other species of eubacteria.

The mechanism governing Y-family polymerases and the regulation of TLS in *E. coli* is known as the SOS response [9,11]. Under normal cellular conditions (*i.e.*, non-stress conditions), a repressor protein inhibits the expression of the Y-family polymerase genes, an idea first proposed by Witkin in 1967 [12]. This mechanism has since been clarified; namely, the repressor protein LexA was identified and shown to bind to operator sites, repress gene expression, and to become inactivated upon DNA damage [11]. When DNA damage is present and replicative polymerases are inhibited, a region of single-stranded DNA (ssDNA) is formed. RecA protein binds to and polymerizes on the newly formed ssDNA, forming a nucleoprotein filament, which is stabilized by the presence of ATP. The LexA repressor protein then binds to the RecA/ssDNA nucleoprotein complex, which induces the autocatalytic cleavage of LexA at the Ala84-Gly85 bond, thereby upregulating the Y-family polymerase genes and others [13,14,15].

Y-family polymerases from *E. coli* and other species of bacteria will be the focus of this review. However, Y-family polymerases are also found in all domains of life, such as archaeal *Sulfolobus solfataricus* Dpo4 [16], *Saccharomyces cerevisiae* Pol η and Rev1 [8], and *Homo sapiens* Pol η, Pol ι, Pol κ, and Rev1 [8,17]. Y-family polymerases share common characteristic structural features such as the palm, finger, and thumb domains [18,19,20,21,22]. Also characteristic of Y-family polymerases is the presence of the little finger domain [23]. The overall size of the finger and thumb domains of Y-family polymerases is smaller than those of their replicative counterparts, which results in an open, solvent‑accessible DNA-binding region to allow for large, bulky lesions to enter the active site [8,24]. In addition, the Y-family polymerases lack intrinsic 3' to 5' exonucleolytic proofreading and lack the characteristic α-helix, known as the ‘O-helix’ in *E. coli* Pol I, which is used in replicative polymerases to improve their fidelity. The lack of this α-helix presumably contributes to the ability of Y-family polymerases to accommodate damaged DNA templates and to their lower fidelity on undamaged DNA [23,25,26,27]. 

## 2. Translesion Synthesis in *E. coli*

### 2.1. UmuD

Prior to discussing *E. coli* Y-family polymerases DinB (Pol IV) and UmuD'_2_C (Pol V), the functions of the *E. coli umuD* gene products will be briefly described, as the *umuD* gene products play critical roles in regulating the activities of both DinB and UmuC. UmuD is the product of one of the genes whose expression is coordinately upregulated along with *dinB* and *umuC* [13,28,29]. The *umuDC* genes are organized in an operon; however, the levels of UmuD appear to correlate more closely with those of DinB than UmuC [30]. Notably, UmuD is not found in all species that have UmuC present, indicating that UmuD is not generally required to regulate UmuC or DinB, although other proteins may fulfill this function in organisms that lack UmuD [8]. UmuD_2_ is the predominant form of the protein for the first 20 to 40 minutes after induction by the SOS response [31]. UmuD, in conjunction with UmuC, acts in a DNA damage checkpoint [32]. When *E. coli* cells are grown at 30 °C and UmuD and UmuC are present at elevated levels, they inhibit DNA replication in a role distinct from their function in TLS [31,33]. They also inhibit the replication process after the cell has been exposed to UV light [31]. When UmuD_2_ interacts with the RecA/ssDNA nucleoprotein filament, the filament facilitates UmuD autocatalytic cleavage, thereby removing the 24 N-terminal amino acids of UmuD to form UmuD' [34,35,36]. UmuD cleavage is similar to the autocatalytic cleavage of LexA, also facilitated by the RecA/ssDNA nucleoprotein filament [37,38,39]. However, the catalytic efficiency of cleavage is much greater for LexA than it is for UmuD_2_[34]. UmuD_2_ cleavage typically occurs about 20 to 40 minutes after the initiation of the SOS response [11,31]. The cleaved form of UmuD_2_, UmuD'_2_, then interacts with UmuC to form UmuD'_2_C (Pol V), which is capable of performing TLS [11,40,41,42]. UmuD' and UmuC prevent RecA-dependent homologous recombination as a result of the interaction between UmuD'_2_C and the RecA/ssDNA nucleoprotein filament [43,44,45]. Full-length UmuD_2_ is involved in prevention of mutagenesis by UmuC or DinB, whereas UmuD'_2_ is involved in facilitation of mutagenesis via Pol V; thus, cleavage of UmuD represents a switch from a non‑mutagenic state to a mutagenic state of a cell [46].

### 2.2. DinB (DNA Pol IV)

DinB, initially identified as the product of the *dinP* gene [47], was discovered in 1980 as one of the DNA *d*amage-*in*ducible genes and was named *dinB* [48]; both names are used in the literature. The *dinB* gene encodes one of the two *E. coli* Y-family DNA polymerases (DinB, or Pol IV) capable of bypassing lesions in DNA via translesion synthesis [49]. DinB is the only Y-family DNA polymerase that is conserved throughout all domains of life, although *S. cerevisiae* apparently lacks a DinB ortholog [8,50]. In non-SOS conditions, DinB is expressed at approximately 250 molecules per cell; however, the number of DinB molecules increases by approximately 10-fold under SOS-induced conditions [51]. During the SOS response, DinB is expressed at the highest level of all five DNA polymerases in *E. coli* [51]. Like other Y-family polymerases, DinB is a DNA-dependent DNA polymerase that is capable of copying damaged primer-template structures and lacks 3'-5' exonuclease proofreading abilities [49]. The fidelity of the *dinB* gene product is lower than that of the replicative polymerase in *E. coli*, namely the Pol III holoenzyme [52]. The presence of DinB during TLS can increase mutagenesis as a result of its relatively low fidelity and the lack of 3'-5' exonuclease activity. [49,51,53,54,55,56]. 

In addition to being upregulated by the SOS response, DinB is involved in a process known as adaptive mutagenesis [57,58]. In an experiment using Lac reporter strains of *E. coli*, there was a seven‑fold decrease in mutations in strains lacking the *dinB* gene compared to in the wild-type strain, suggesting that DinB can induce mutations [57]. These mutations, which result from the adaptive mutagenesis process, can cause cells to have a selective advantage during stressful conditions [59]. The precise mechanism for adaptive mutagenesis and how it leads to high levels of DinB expression is not fully understood [60,61,62], although one mechanism may be through DinB involvement in error‑prone double-strand break repair [63]. Overall, DinB expression can be considered a general stress response mechanism that can lead to high rates of mutagenesis and could ultimately result in antibiotic resistance, as described below [57,64,65,66]. Moreover, DinB contributes to cellular fitness and long‑term survival in stationary phase [67]. DinB also has a non-catalytic function, in that elevated levels of DinB slow the replication fork in a checkpoint-like phenomenon [68,69,70]. 

Currently, no crystal structure of DinB has been solved; however, homology models [71,72,73] have been constructed using the crystal structures of Dpo4 from *Sulfolobus solfataricus* [23] and Dbh from *Sulfolobus acidocaldaricus* [16] as templates. These models allow for the prediction of specific residues involved in DinB function. For example, the steric gate residue, which is the amino acid residue of a DNA polymerase that prevents ribonucleotides from entering the active site [74,75], of DinB is F13 [73]. Changing the steric gate residue of DinB increases the frequency of ribonucleotide incorporation from less than 10^−5^ to 10^−3^, as well as increases the ability of DinB to replicate undamaged DNA [73].

DinB is known to bypass certain dG adducts (Table 1). For example, DinB bypasses *N*^2^-furfuryl dG [73], *N*^2^-benzo[a]pyrene-dG [72,76], *N*^2^-(-1-carboxyethyl)-dG [77], *N*^2^-*N*^2^-dG interstrand crosslinks [78], and γ-hydroxypropano-dG [79]. DinB is effective in bypass of *N*^2^*-*dG adducts formed from benzo[a]pyrene (B[a]P), a bulky polycyclic carcinogen which is metabolically activated to form 7*R*,8*S*-dihydroxy-9*S*,10*R*-epoxy-7,8,9,10-tetrahydrobenzo[a]pyrene [72,76]. In the presence of DinB, *N*^2^*-*B[a]P-dG adducts are bypassed with relatively high fidelity and efficiency with a misincorporation frequency of 10^−2^ to 10^−4^ [76]. In addition, DinB has been shown to accurately and efficiently bypass *N*^2^-(1-carboxyethyl)-2'-deoxyguanosine (*N*^2^-CEdG) adducts [77]. *N*^2^-CEdG minor groove adducts are formed endogenously from methylglyoxal, which is a byproduct of glycolysis [80,81], and are detected at one lesion per 10^7^ nucleotides in human melanoma cells [80]. 

An *E. coli* strain containing a deletion of the *dinB* gene is sensitive to both nitrofurazone (NFZ) and 4-nitroquinoline-1-oxide (4NQO) [73]. Both of these DNA-damaging agents are thought to form *N*^2^‑dG adducts *in vivo* [82,83]. DinB shows greater accuracy and 15-fold increased proficiency of dCTP insertion opposite *N*^2^-furfuryl-dG, an *N*^2^-dG adduct likely formed from NFZ, than opposite undamaged DNA [73]. DinB has also been shown to accurately bypass *N*^2^-*N*^2^-dG interstrand crosslinks (ICLs), which can disrupt DNA replication [78]. Interstrand crosslinks are typically repaired by cooperation between homologous recombination and nucleotide excision repair [84], but recent work has shown that TLS by DinB may also play a role in repair of ICLs [78]. The γ-hydroxypropano-dG adduct, as well as other adducts formed from α,β-unsaturated aldehydes, can form DNA-peptide crosslinks [79]. DinB has been shown to bypass these acrolein-mediated adducts as well as the interstrand crosslinks and the peptide conjugates that form from these adducts [79,85]. 

**Table 1:** Comparison of adducts bypassed *in vitro* and *in vivo* by DinB or UmuD'_2_C. Relevant references and abbreviations are listed in the text.

DNA polymerase	*in vivo*	*in vitro*
**DinB (Pol IV)**	*N*^2^-furfuryl-dG (the presumed adduct formed from nitrofurazone); *N*^2^-B[a]P-dG; *N*^2^-CE-dG; ICLs; adducts formed from reactive oxygen species	*N*^2^-furfuryl-dG; *N*^2^-B[a]P-dG; *N*^2^-CE-dG; ICLs; 8oxodG, 2oxodA, 5-fodU, hmdU (adducts formed from reactive oxygen species); abasic sites
**UmuD** **'_2_C (Pol V)**	Abasic site; T-T CPD; T-T (6-4) photoproduct; *C*^8^-AAF; adducts formed from oxidized dG	Abasic sites; T-T CPD; T-T (6-4) photoproduct; *C*^8^-AAF; *N*^2^-B[a]P-dG; *N*^6^-B[a]P-dA

DinB confers resistance to the alkylating agent methyl methanesulfonate (MMS) [86]. A cluster of DinB residues referred to as the ‘aromatic triad’, F12, F13, and Y79, is important for survival of *E. coli* cells in the presence of MMS [87]. *E. coli* strains that contain single-point mutations in the ‘aromatic triad’ residues within DinB show fewer MMS-induced mutations than nitrofurazone-induced mutations, which suggests that these residues not only play a significant role in TLS, but also are involved in modulating the accuracy of DinB in bypassing specific lesions [87].

DinB is also involved in bypass of lesions that result from various reactive oxygen species leading to A:T → G:C transitions [88]. In studies with defined lesions, DinB was shown to preferentially insert dATP opposite 5-formyluracil (5-fodU) and 5-hydroxymethyluracil (5-hmdU); both dCTP and dATP opposite 7,8-dihydro-8-oxoguanine (8-oxo-dG) with low efficiency; and both dCTP and dTTP opposite 1,2-dihydro-2-oxoadenine (2-oxo-dA) with dCTP inserted more efficiently [88]. In addition, DinB was found to incorporate 8-hydroxy-dGTP opposite both adenine and cytosine and 2-hydroxy-dATP opposite both guanine and thymine *in vitro* [89,90]. Oxidation of dGTP to 8-oxo-dGTP is the cause of cell death that results from treatment with antibiotics and from elevated levels of DinB, due to increased incorporation of 8-oxo-dGTP by DinB [89]. The evidence supports a model in which cytotoxicity results from double strand breaks caused by incomplete repair of 8-oxo-dG lesions that are closely spaced [89]. 

It has been further shown that DinB is capable of adding dGTP opposite the modified pyrimidine 1,3-diaza-2-oxophenothiazine (tC) specifically but is incapable of continuing TLS beyond the modified base [91]. This is intriguing since DinB binds slightly more strongly to DNA primer/template constructs that contain the tC analog than it binds to an undamaged DNA primer/template, which may indicate a specific inability to bypass major-groove modified bases in DNA [91]. However, it was also found that DinB inserts tC opposite G in the DNA template and is capable of extending from the newly-incorporated tC, suggesting that DinB shows asymmetric discrimination against the modified DNA template and the incoming nucleotide [91]. 

The error frequency of DinB on undamaged DNA is approximately 2.1 × 10^−4^ for generating frameshift mutations and about 5.1 × 10^−5^ for generating base substitution mutations [52]. DinB is also known to bypass abasic sites, causing −1 frameshift mutations [52,92]. One model for this is a ‘dNTP‑stabilized’ misalignment mechanism in which dNTP is placed correctly opposite a template base downstream rather than placing an incorrect nucleotide opposite the next available template base [52]. More recently however, evidence for another mechanism involving template slippage has been observed, which provides another possible explanation for the generation of −1 frameshift mutations [93]. The template slippage mechanism causes single base deletions on DNA containing homopolymeric runs [93]. When UmuD_2_ is bound to DinB, a non-slipped conformation is preferred which prevents the generation of frameshift mutations [93].

It is known that the following proteins interact directly with DinB and affect its replication efficiency: UmuD_2_, RecA, NusA, Rep helicase, single-stranded DNA binding protein (SSB), and the β-processivity clamp subunit [30,94,95,96,97,98]. The presence of UmuD and RecA improves the catalytic efficiency of DinB and also reduces the number of −1 frameshift mutations generated by DinB *in vitro* [30]. Deletion of the *umuD* gene leads to an increase in the frequency of −1 frameshift mutations; however, the resistance of such cells to nitrofurazone was not affected, suggesting that the ability of DinB to perform TLS and the mechanism for generating −1 frameshift mutations are separable functions [30]. 

The C-terminus of DinB (residues 347–351) binds to the β-clamp subunit of the Pol III holoenzyme; this interaction contributes substantially to both enzyme processivity in TLS and dNTP-binding affinity [94,99,100,101]. A second binding site has also been observed between residues 303–305 of the DinB little finger domain and residues located near the dimer interface of the β-clamp subunit [100]. Binding of DinB to the β-clamp subunit increases the processivity of the polymerase, helps to position DinB correctly at the replication fork, and coordinates polymerase usage [100,102,103,104,105,106,107].

NusA, which has roles in elongation, termination, and anti-termination of transcription [108,109,110], has also been shown to interact with DinB. NusA recruits DinB to gaps in the DNA template strand during transcription-coupled TLS when RNA polymerase is stalled by a lesion in DNA [95,96]. NusA has been shown to be necessary for stress-induced mutagenesis by DinB [111]. The exact binding site of DinB on NusA is unknown, but it is believed to be located in the C-terminal domain of NusA [95,112]. The NusA-DinB interaction is predicted to bridge the gap between replication-coupled TLS and transcription-coupled TLS [96]; therefore, this work demonstrates a crucial connection between replication and transcription, especially in the presence of DNA damage. 

### 2.3. UmuD'_2_C (DNA Pol V)

*E. coli* Pol V is the second of the two Y-family polymerases found in *E. coli*. Pol V consists of two different protein subunits, one UmuD'_2_ dimer and UmuC, which interact to form UmuD'_2_C [42,113,114,115]. The *umuC* gene was discovered in the late 1970s to be required for *E. coli* cells exposed to UV light to mutate [114]. The ability of UmuC to bypass UV-induced DNA adducts via TLS was not discovered until well after UmuC was characterized as being involved in SOS mutagenesis. Several models were proposed over the course of the next few decades to explain the role of UmuC in mutagenesis [116,117,118,119]; however in the late 1990s, it was determined that UmuC was in fact a DNA polymerase [40,41,42,120]. This was confirmed when it was shown that UmuC exhibited low fidelity lesion bypass on its own, but its fidelity and efficiency increased in the presence of RecA, SSB, and UmuD' [40,120,121]. In a key finding, UmuC maintained its ability to function even in the absence of the Pol III holoenzyme [40,120]. 

Similar to the SOS regulation of *dinB* expression, the genes encoding the protein constituents of Pol V, *umuC* and *umuD*, are both regulated by the SOS response but the *umuD* and *umuC* genes are located within the same operon [28,29,122]. Also similar to DinB, upon induction of the SOS response, the expression of the Umu proteins increases 10-fold, with UmuC increasing from approximately 15 to 200 molecules and UmuD increasing from approximately 180 to 2,400 molecules [123]. DNA repair processes such as nucleotide excision repair typically remove a lesion once it has formed in DNA [124,125]; however, in the event that nucleotide excision repair does not take place, replication will recover upon induction of Pol V [125]. It has been shown that when the *umuC* gene is deleted, there is moderate DNA synthesis recovery and when the *recJ* gene is deleted, there is poor recovery of DNA synthesis after damage [126]. When both the *umuC* and *recJ* genes are deleted, there is no recovery of DNA synthesis after damage, indicating that *recJ* and *umuC* both act to restore stalled replication forks [126]. 

The ability of Pol V to perform TLS is dependent on the formation of the UmuD'_2_C complex and the presence of RecA [40,127]. Full-length UmuD is involved in preventing UmuC from engaging in TLS and therefore preventing mutagenesis by UmuC. Changing the active site Ser60 residue to Ala in full-length UmuD prevents autocatalytic cleavage of UmuD_2_[35]. Full-length UmuD is involved in prevention of mutagenesis; thus it was found that UmuD_2_ harboring the S60A mutation results in a large reduction of UV-induced mutagenesis [31,128,129]. Cells that contain UmuD_2_-S60A with UmuC experience greater sensitivity to UV light relative to cells that contain wild-type UmuD and UmuC [35,128,129]. Molecular interactions between UmuC and UmuD have been difficult to determine; however, through immunoprecipitation, glycerol gradient analysis, and yeast two hybrid assays, the physical interaction of UmuD' and UmuC was confirmed [115,130]. The interaction of full-length UmuD and UmuC was also confirmed using affinity chromatography and velocity sedimentation analysis [115]. The physical interaction between UmuD' and UmuC consists of one UmuC protein bound to a dimeric UmuD' [115], with UmuD' interacting with the 25-amino acid C‑terminal end of UmuC [32,115]. 

To date, there is no experimentally-determined structure of UmuC. Homology models have been constructed of the polymerase and little finger domains [24,128,131], but as the C-terminal domain possesses little homology to proteins of known structure, a model of the entire protein cannot be constructed at this time. Still, the model has allowed specific predictions of the functions of particular residues to be tested. In particular, the steric gate residue (Y11) [131,132,133], hydrophobic residues (I38, A39) near the nascent base pair [134], and a cluster of residues (N32, N33, D34) near the incoming nucleotides have all been shown to contribute to UmuC function [135]. 

In general, Pol V is capable of bypassing lesions formed in DNA that DinB is incapable of bypassing. Pol V can bypass lesions caused by exposure to UV light such as thymine-thymine (T-T) *cis-syn* cyclobutane pyrimidine dimers (CPD) and T-T (6-4) photoproducts (Table 1) [101,105,106,136]. In addition to bypassing lesions from UV light, Pol V is capable of bypassing abasic sites, *C*^8^-dG lesions such as *N*-2-acetylaminofluorine (*C*^8^-AAF), as well as being required for replicating DNA containing 5'*S*-8,5'-cyclo-2'dG [92,101,105,106,136,137]. Pol V bypasses lesions caused by UV light with greater efficiency when in the presence of the β-clamp subunit, the RecA/ssDNA nucleoprotein filament, and SSB [136]. Pol V can be mutagenic when carrying out TLS. For example, Pol V inserts dGTP opposite the 3'T in T-T (6-4) photoproducts instead of dATP with a six-fold greater frequency [136]. However, Pol V is also known to bypass certain lesions with high accuracy. For instance, Pol V faithfully inserts dATP opposite both Ts of T-T CPDs [136] as well as dCTP opposite *C*^8^-AAF-dG [106]. Pol V also bypasses *N*^2^-benzo[a]pyrene-dG adducts, *N*^6^-benzo[a]pyrene-dA adducts and adducts resulting from oxidation with varying accuracy [72,76,138]. Pol V replicates undamaged DNA with error frequencies of 10^−3^ to 10^−4^, which is a much lower fidelity than the Pol III holoenzyme and a lower fidelity than DinB [136]. It should be noted that Y-family DNA polymerases can be accurate or error-prone depending on the cellular and DNA context in which they are acting. 

In addition to facilitating the autocatalytic cleavage of the LexA repressor protein as well as facilitating the cleavage of full-length UmuD_2_ to UmuD'_2_, RecA/ssDNA nucleoprotein filaments also play a direct role in TLS [40,41,139,140,141]. RecA has been determined to stimulate both nucleotide insertion and extension [141]. One model suggests that Pol V and two RecA molecules form a complex that activates Pol V for TLS in the presence of ATP [127,142]. There is evidence that a distinct RecA/ssDNA nucleoprotein filament transfers RecA and an ATP from the 3' end of this *trans* filament to Pol V, which activates Pol V for TLS [143,144]. However, other work suggests that the RecA/ssDNA nucleoprotein filament acts in *cis* on DNA directly downstream of pol V rather than *in trans* to facilitate the activation of Pol V and TLS [107]. These differences are likely the result of differences in experimental details. The complex of activated RecA with UmuD'_2_C is termed the Pol V mutasome [127].

In addition to RecA, the β-processivity clamp and the γ clamp loader increase the processivity of Pol V by allowing the enzyme to remain bound to the DNA and by providing additional stability [145]. The β clamp increases processivity approximately three-fold to five-fold in one study [146] and about 100-fold in another study [107]. Clearly, the β clamp stimulates processivity of Pol V, but the extent of the increase in processivity depends on the specifics of the experimental system [19].

### 2.4. E. coli Translesion Polymerases in Antibiotic Resistance

As both DinB and Pol V can be mutagenic depending on the cellular context and the nature of DNA lesions encountered, it was speculated that they could contribute to antibiotic resistance [64,65,66]. Indeed, in *E. coli* cells grown under stress conditions, DinB and to a lesser extent Pol V are responsible for base substitution mutations in the *ampD* gene that result in resistance to the β-lactam antibiotic ampicillin [147]. Moreover, DNA Pols II, IV, and V all contribute to *E. coli* resistance to the antibiotic ciprofloxacin, as well [148]. While the contribution of each polymerase was examined in bacterial cultures, in a mouse model of infection with pathogenic *E. coli*, LexA cleavage was required for resistance to ciprofloxacin or rifampicin [148]. Thus, SOS-regulated DNA polymerases and possibly other genes under LexA control contribute to the evolution of antibiotic resistance in bacteria both when they are grown in the free-living state and during infection within a mammalian host. 

### 2.5. E. coli Translesion Polymerases in Pathogenesis

A recent study has shown that DinB of uropathogenic *E. coli* (UPEC) is required for virulence of UPEC strains in bladder infections in mice [149]. Deletion of *dinB* in all UPEC isolates tested results in a reduced ability to colonize host bladders. No reduction in virulence of the *dinB*-deletion mutant is observed in mice that have a reduced Toll-Like Receptor-4 (TLR4)-dependent inflammatory response, indicating that DinB is important in helping UPEC cope with the stresses produced by host inflammation. In contrast, deletion of *umuDC* does not reduce the virulence of these UPEC strains. Surprisingly, cells of the *dinB*-deletion mutant recovered from the host have a mutation frequency similar to that of the wild-type parent. This is in contrast to the phenotype observed in culture, in which the *dinB*-deletion mutant of the UPEC strain UTI89 has a reduced rate of spontaneous mutation when grown in either rich medium or in human urine. This study demonstrated a clear role for DinB in UPEC pathogenesis and virulence. However, DinB does not appear to influence the acquisition of mutations by UPEC in the host environment.

## 3. DinB and UmuC Orthologs in Other Bacteria

Although the Y-family polymerases of *E. coli* have been well studied, Y-family polymerases are present throughout all domains of life [8]. The following will discuss recent developments in the study of Y-family polymerases in other species of eubacteria. The close relative of *E. coli*, *Salmonella typhimurium,* possesses homologs of DinB, UmuD, UmuC, and a second pair of UmuD and UmuC homologs, known as SamA and SamB [150]. A *S. typhimurium* strain lacking DinB and both Pol V homologs had a sharp reduction in the frequency of spontaneous deletion formation [150]. Conversely, a strain overproducing Pol IV (but not the Pol V homologs) has an increased frequency of spontaneous deletions [150]. *Acinetobacter* species have a range of configurations of *umuD*, *umuC*, and *dinB* genes [151]. For example, *Acinetobacter baylyi* possesses a *umuD* gene with a 5' extension but only fragments of *umuC* [152], whereas *Acinetobacter ursingii* harbors the extended version of *umuD* in an operon with *dinB* [151]. Moreover, despite the presence of *umuD-*, *umuC-*, and *dinB-*like genes in *Acinetobacter* species, UV-induced mutagenesis was observed in only a few of the species tested, including in the opportunistic pathogens *Acinetobacter baumanii* and *Acinetobacter ursingii* [151]. 

Y-family polymerases in the bacterium *Bacillus subtilis* have been demonstrated to be involved in mutagenesis [153]. The Y-family polymerases encoded in the *B. subtilis* genome are UvrX, YqjH, and YqjW, which have significant homology to the *E. coli* Y-family polymerases DinB and UmuC [8]. The *B. subtilis* genome sequence data indicates that UvrX is encoded in the prophage known as SPβ [154]. UvrX has 25% sequence identity to *E. coli* DinB and is involved in repair of UV damage [154]. The constitutive YqjH protein has 36% sequence identity to *E. coli* DinB and the SOS-inducible YqjW protein has 26% sequence identity to *E. coli* UmuC [155]. Inactivation of the *yqjH* and *yqjW* genes results in increased UV sensitivity and decreases the frequency of UV-induced mutagenesis [156]. The lack of a UmuD homolog in *B. subtilis* could indicate that another protein is fulfilling its function of regulating mutagenesis. Recently, it has been found that YqjH and YqjW are involved in protecting sporulating cells of *B. subtilis* [157]. Deletion of *yqjH* and *yqjW* genes decreases sporulation efficiency as well as increases sensitivity to chemical mutagens such as hydrogen peroxide, *tert*‑butylhydroperoxide, mitomycin C (MMC), and UV-C radiation [157]. It was concluded that YqjH and YqjW proteins are involved in TLS in sporulating *B. subtilis* cells and cause spontaneous mutations [157].

*Mycobacterium tuberculosis* contains two Y-family DNA polymerases, both of which are homologous to *E. coli* DinB. They are identified as DinB1 (or DinX), which is encoded by the gene *Rv1537*, and DinB2 (or DinP), which is encoded by the gene *Rv3056* [158]. These proteins possess sequence similarity to their homologs in *E. coli* [49] as well as to those in *Pseudomonas aeruginosa* [159], leading to the presumption that DinB1 and DinB2 both have DNA polymerase activity. Unlike Y-family polymerases from *E. coli* and most other eubacteria, expression of DinB1 and DinB2 does not depend on the RecA protein, the SOS response, or even the existence of damaged DNA [160,161,162,163]. In contrast, DinB1 and DinB2 are regulated by separate mechanisms whereby DinB1 is expressed in pulmonary tuberculosis [164] and DinB2 is expressed upon exposure to novobiocin [160]. While this work determined that the DinB homologs in *M. tuberculosis* are not induced upon DNA damage as in other organisms, the C-family DNA polymerase DnaE2 was induced by the presence of DNA damage (see also Sections 4 and Sections 7.1) [165]. The C-family DNA polymerases were previously considered high‑fidelity, replicative DNA polymerases in bacteria; however, the C family includes a subfamily of polymerases, including DnaE2, the members of which are capable of carrying out TLS [166]. DnaE2, rather than the DinB homologs, was therefore predicted to play the primary role in adaptive mutagenesis in *M. tuberculosis* [165]. 

The bacterium *Mycobacterium smegmatis* also contains homologs of *E. coli* Y-family polymerases. It was found that the genome of *M. smegmatis* contains three DinB homologs encoded by the genes *msmeg_1014*, *msmeg_3172*, and *msmeg_6443* according to the KEGG PATHWAY Database [167]. Interestingly, the key residues necessary for functional polymerase activity are conserved in *msmeg_1014* (also known as MsDpo4) [168]. MsDpo4 is capable of performing template-dependent nucleotide insertion and can promote mismatches on undamaged DNA templates [168]. In addition, MsDpo4 has been shown to preferentially promote G:T and T:G mismatches, indicating that it has the ability to increase the frequency of untargeted mutations [168].

Y-family DNA polymerase homologs are also present in species of the bacterial genus *Pseudomonas* [8]. *Pseudomonas aeruginosa* contains a homolog of *E. coli* DinB (PaDinB), which is lacking in intrinsic proofreading capabilities [159]. PaDinB promotes C to A transversions as well as induces −1 frameshift mutations [159]. Strains lacking the *dinB* gene are sensitive to the DNA‑damaging agents nitrofurazone (NFZ) and 4-nitroquinoline oxide (4NQO) showing that PaDinB most likely plays a role in TLS similar to that of *E. coli* DinB [159]. On the other hand, PaDinB accurately copies TT CPDs but contributes to H_2_O_2_-induced mutagenesis [169]. A DinB homolog was also found in *Pseudomonas putida* and was shown to be involved in 1-base pair (bp) deletions in starving cells, yet was also reported to be expressed in a RecA-independent process [170]. *P. putida* also possesses a plasmid‑borne homolog of Pol V that confers resistance to DNA damage, increases fitness, and whose expression is regulated in a RecA-dependent manner [171]. 

## 4. The Function of *dnaE* and Discovery of a Second *dnaE* Gene

In most bacteria the main replicative polymerase is the C-family DNA polymerase DnaE (α subunit) or PolC [172,173], which is the polymerase subunit of the DNA Pol III holoenzyme, a complex of 10 different subunits [166]. In some organisms such as *E. coli*, there are two or three copies of DnaE in the Pol III holoenzyme, which is encoded by a single gene, *dnaE* [166,174,175]. In other organisms such as *B. subtilis* [176], one α subunit is encoded by *dnaE* and another α subunit is encoded by *polC*, each of which has a distinct role corresponding to DNA synthesis on the leading and lagging strands in the replication process [166]. Notably, *B. subtilis* DnaE is SOS-inducible and is capable of TLS [177]; similarly *Streptococcus pyogenes* DnaE is error-prone and can carry out TLS [178]. 

As early as 1995, it was noted that various *Mycobacterium* and *Mycoplasma* species contained an extra *dnaE* gene [158,179,180,181], which due to the lack of an identifiable 3'-5' exonuclease domain were characterized as another α subunit gene. This extra *dnaE* gene resulted in the primary, replicative gene to now be designated *dnaE1* (for example, Rv1547c in *Mycobacterium tuberculosis*) and the extra copy designated as *dnaE2* (Rv3370c in *M. tuberculosis*).

In some cyanobacteria the *dnaE* gene products are DnaE1 and DnaE2, which are split by inteins and combine to form the intact PolC [182], compounding the number of different “*dnaE2*”s in the literature. In order to deal with the proliferation of *dnaE* relatives, a new system of nomenclature has been proposed [166]. The *dnaE2* gene, referring to a homolog of *E. coli dnaE* that is not essential for replication, is often found following or accompanied by the two genes *imuA* and *imuB*, and thus *dnaE2* is now referred to as *imuC* (Table 2)[166].

**Table 2:** Summary of DNA polymerases and their accessory factors. For each protein, a species that contains the most studied or most representative protein is listed. Especially for newly described mutagenesis cassettes, the roles of these proteins are still uncertain or incomplete.

Protein	Role	Representative species
DinB	Bypass of *N*^2^-dG adducts, −1 frameshift mutagenesis	*E. coli*
UmuD'_2_C	Bypass of UV-induced lesions, induced mutagenesis	*E. coli*
SamAB	Plasmid-borne UmuDC homologs	*S. typhimurium*
UvrX	UV damage repair, sporulation	*B. subtilis*
YqjH	UV damage repair, sporulation	*B. subtilis*
YqjW	UV damage repair, sporulation	*B. subtilis*
ImuC	Induced mutagenesis from UV/MMC	*M. tuberculosis*
ImuB	Binds processivity factor; role in polymerase switching?	*M. tuberculosis*
ImuY	Same pathway as *D. deserti* ImuC, analogous to ImuB?	*D. deserti*
ImuA	Mostly unknown, found in species with ImuB/ImuC	*C. crescentus*
ImuA'	Mostly unknown, interacts with ImuB	*M. tuberculosis*

## 5. Discovery of Associated Genes *imuA*, *imuA'*, *imuB*, *imuY*

In *Pseudomonas putida*, reverse transcriptase PCR (RT-PCR) showed multiple genes in a cassette annotated as *lexA2*, *sulA*, *dinP* (by analogy to *E. coli dinP* or *dinB*), and *dnaE* are expressed under the direct control of the *lexA2* gene as a single transcription unit [183]. Phylogenetic analysis showed a widespread occurrence of this mutagenic cassette. The cassette is unlikely to have been acquired recently by these genomes, given the similar GC content of each cassette with that of its genomic environment [183]. The *sulA*, *dinP*, and *dnaE* genes were all determined to be involved in DNA replication and mutagenesis [183]. Subsequently, the original annotations of *sulA*, *dinP*, and *dnaE* were changed to *imuA*, *imuB*, and *imuC*, respectively, to reflect the re-classification of these genes as encoding novel proteins [183,184,185]. The names of *imuA* and *imuB* are derived from “*i*nducible *mu*tagenesis” [186]. As the third gene in the cassette, *dnaE2* was proposed to be renamed *imuC* as the logical extension of the names of the genes in the operon [166,187].

While the ImuB amino acid sequence is closely related to those of Y-family polymerases, the amino acids that correspond to catalytic aspartic acids in most other Y-family polymerases are missing, and thus this protein is thought to be biochemically inactive in translesion synthesis [187,188]. ImuA and its homolog in *M. tuberculosis*, ImuA', share some sequence similarity with LexA, RecA, and SulA, but little is known about the function of the ImuA' proteins. *Bdellovibrio bacteriovorus imuA* is not able to complement an *E. coli recA^−^* mutant [189]. In some organisms, prominently in the Actinomycetales, such as *Mycobacterium tuberculosis*, the *imuA* candidate gene is so dissimilar from the proteobacterial *imuA* that the *M. tuberculosis* gene has been named *imuA'* to mark its notable difference [190]. Similarly, *imuY* in *Deinoccocus deserti* [191,192] lacks similarity to known *imuB* genes, and since it was implicated in translesion synthesis with a Y-family polymerase-like sequence, was termed *imuY* [193].

## 6. *imuABC* Operon Regulation and Organization

Expression of the *imuA*, *imuB*, and *imuC* genes is almost exclusively controlled by LexA-SOS systems. Many of the genes in the *imuABC* family were discovered during searches for SOS-box-containing LexA binding motifs. The *imuABC* genes were found following the SOS boxes, closely linking the discovery of LexA binding motifs and *imuABC* genes. Elucidating the evolutionary history of LexA binding motifs can be difficult due to the short sequence of the SOS box [184]. With the increasing number of *imuABC* gene sequences now known, the identification of putative *imuABC* genes can be utilized to augment phylogenetic analysis of the LexA binding motifs [190]. In organisms with a recognizable SOS box motif, *imuABC* open reading frame(s) provide an opportunity to track the SOS-box with much higher precision than the SOS box alone.

In *P. putida*, there are two different LexA regulons controlled by LexA1 and LexA2 [184]. It was shown that LexA2 directly controls the *lexA2*-*imuA*-*imuB*-*imuC* operon as a single transcriptional unit when induced by MMC in *P. putida* [183]. There seems to be evolutionary pressure to include LexA regulation of the *imuABC* mutagenic cassette once an organism has acquired at least *imuB* and *imuC* [190]. 

**Figure 1:**
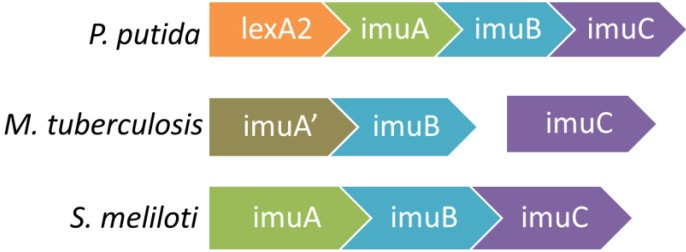
Some characteristic configurations of the *imuABC* mutagenesis cassette are shown below. The plethora of *imuABC* operons has been characterized extensively [183,190,195]. While *P. putida* and *S. meliloti* have *imuA*, *imuB*, and *imuC* together as one operon, *M. tuberculosis* contains *imuC* separated from *imuA* and *imuB*, as well as *imuA*' instead of *imuA*.

The *imuABC* genes are not found in cyanobacteria or gram-positive bacteria. A complete mutagenesis cassette is often found in the form of a single operon *imuA-imuB-imuC* such as in many *Alphaproteobacteria* (Figure 1). For some *Alphaproteobacteria* such as *Sinorhizobium meliloti* and *Agrobacterium tumefaciens*, not only are their cassettes a single uninterrupted operon, cassettes can be found on both the chromosome as well as on plasmids [190]. In *Ralstonia solanacearum*,the cassette exists only on its plasmid [183,194].

Members of the *imuABC* cassette can be organized as an uninterrupted *imuABC* operon, or in other configurations where various members are found in different loci or with members missing. A lone *imuC* gene is found in some bacteria, such as *Kineococcus radiotolerans*, *Symbiobacterium thermophilum*, and *Actinomyces naeslundii*, while an *imuBC* cassette is found in *Streptomyces coelicolor* [190]. *M. tuberculosis*, which has one of the most thoroughly studied *imuABC* systems, has a configuration with *imuA*' and *imuB* together at one locus and *imuC* at another locus, both of which are controlled by LexA [190]. Almost all configurations exist in different bacteria, including each of the *imuA*, *imuB*, and *imuC* genes located at a distinct locus each with their own SOS box [190,195].

## 7. Known Functions of the Mutagenesis Cassette Gene Products

### 7.1. Mycobacterium

When identifying the SOS boxes of *Mycobacterium tuberculosis* and the induction levels of the genes proposed to be *lexA* regulated, Davis *et al*. found a gene annotated as *dnaE2* (*i.e.*, *imuC*) with a preceding *M. tuberculosis* LexA-binding SOS box [196]. On average, the Rv3370c (*imuC*) gene was up-regulated more than 10-fold following induction by MMC [196]. It has been shown that MMC induces *imuC*, *recA*, and *lexA* in strains containing a functional RecA, but that *imuA'B* and *imuC* are not induced by MMC in a *recA-*deletion mutant [162,197]. An *imuC* null mutant of *M. tuberculosis* has a reduced virulence relative to that of the wild type and is deficient in UV-induced mutagenesis [165]. This experiment showed that *imuC*-mediated mutagenesis is the sole source of UV-induced mutagenesis in *M. tuberculosis*, with a mutational spectrum that resembles that of a signature for translesion synthesis [165]. Strains with *imuC* reproducibly generated CC to TT mutations, consistent with bypass of a UV damage-induced pyrimidine dimer, whereas in strains without *imuC*, this mutation was not observed [165]. Overexpression of *imuC* in non-UV-treated cells does not increase the mutation frequency, suggesting that *imuC* requires additional subunits to function [165].

In *M. tuberculosis*, *recA* controls expression of *imuA*', *imuB*, and *imuC* [188,198]. MMC also induces the SOS response in *M. smegmatis*. Loss of *imuA*', *imuB*, or *imuC* individually or in combination results in the same level of hypersensitivity to MMC, suggesting that the products of these genes function as part of a single pathway for resistance to MMC [188]. *M. tuberculosis imuC* has three aspartic acids that correspond to the known active site acidic residues of C-family Pol III polymerase catalytic subunits. The *M. smegmatis*^441^DID^443^ to ^441^AIA^443^ mutation, which changes two of the three conserved active site residues, eliminates UV-induced mutagenesis and confers on *M. smegmatis* hypersensitivity to MMC, mimicking the *imuC* deletion phenotype [188]. These experiments established strong evidence that *imuC* is responsible for induced mutagenesis and survival under DNA-damage stress conditions via translesion synthesis [188]. 

An extensive study by Warner *et al*. elucidated many of the interactions between the *imuA*, *imuB*, and *imuC* gene products [188]. Whereas ImuC lacks a β-binding motif to interact with the processivity factor at the replication fork, ImuB does contain a β-clamp-binding motif. Only ImuB interacts with DnaE1 or with the β-processivity clamp. ImuA' and ImuC showed interactions with only ImuB and not with each other or with the β clamp [188]. The ImuB-β interaction can be disrupted by mutation of the β-binding motif or by truncation of the C-terminal end of ImuB including the β-binding motif. Truncations up to, but not including, the β-binding motif of ImuB did not disrupt the interactions with ImuA', ImuC, or β [188]. Truncation of the C-terminal 44 residues of ImuB disrupted the ImuA'-ImuB interaction [188]. Thus, each of the three proteins expressed from the *imuABC* operon interact in a pairwise fashion with ImuB, which leaves open the possibility of ternary complex formation with ImuB occupying a central position in such a ‘mutasome’. 

### 7.2. Deinoccocus

In *Deinococcus deserti*, which was isolated in the Sahara desert [191,192], the genome contains a mutagenesis cassette in the form of *lexA*-(*imuB*-like protein)-*imuC*, as a single transcriptional unit [193]. This operon has unusual characteristics compared to other *imuABC* cassettes. The *imuB*-like gene is different enough from other *imuB*s that the gene in *D. deserti* was termed *imuY*, to recognize its homology to Y-family DNA polymerases, rather than *imuB* [193]. In addition there is a hypothetical protein of 243 base pairs between *imuY* and *imuC* [193]. *D. deserti* has three *recA* genes encoding two RecA products: *recA_C_*, encoding chromosomal RecA_C_, and *recA_P1_* and *recA_P3_*, which both encode the same plasmid-derived RecA_P_ product [193]. The mutagenesis cassette was induced by RecA_C_ but not by RecA_P_ [193]. Interestingly, while transcriptional regulation of the cassette was dependent on RecA_C_, *recA_C_* mutants did not show a loss in UV or gamma radiation survival [193]. Deletion of *imuY*, *imuC*, or both *imuY*-*imuC* showed the same 10-fold decrease in UV-induced mutagenesis, and deletion of *imuY* could be complemented by a plasmid carrying the *imuY* gene [193]. To explain the lack of decreased survival upon *imuY* or *imuC* deletion, Dulermo *et al.* note that the conditions in the native environment of the Sahara desert starkly differ from the mild conditions in the laboratory; under the combined stress of dessication, starvation, and other environmental conditions, *D. deserti* may depend more heavily on *imuY* and *imuC* for survival [193]. 

In *Deinococcus ficus*, a *lexA*-*imuB*-*imuC* gene cassette and a *dinB2* gene are carried on an accessory plasmid [199]. Disruption of either *imuB* or *imuC* showed equal loss of survival and loss of mutagenesis following UV exposure, suggesting the same pathway of action for both survival and induced mutagenesis [199]. *Deinoccocus ficus* naturally possesses keratinolytic activity to break down feathers, which could be used in agricultural and industrial applications [199]. Using the inherent mutator properties of ImuC, UV exposure was utilized as a mutagen to create improved keratinolytic activity [199]. Induced mutagenesis by UV light led to at least one mutant strain with a two-fold higher activity [199]. Zeng *et al*. suggest that increased keratinolytic activity after UV exposure could come from *imuBC*-dependent induced mutations, but note the possibility of the two *dinB* genes to contribute to this process [199]. In addition, *D. grandis* contains a putative *imuB* showing high similarity to the *D. ficus imuB* [199].

The two most thoroughly studied *Deinoccocus species*, *D. radiodurans* and *D. geothermalis*, do not contain mutagenic or translesion synthesis polymerases [193]. It has been hypothesized that it is advantageous for *Deinoccocus* species not to have error-prone translesion synthesis or mutagenic polymerases in order for them to accomplish their striking feats of DNA repair [200]. The discovery of a mutagenic cassette in *D. deserti* and *D. ficus* provide an interesting example of how different species within the same genus can develop different survival strategies [193,199]. 

### 7.3. Caulobacter

In *Caulobacter crescentus*, *imuABC* genes are responsible for UV- and MMC-induced mutagenesis [186] and are strongly repressed by LexA, with increased expression in a *lexA* mutant strain by 15-fold over that of the wild type [201]. While deletion of a single gene in the mutagenic cassette results in slight sensitivity to UV exposure and abolishes induced mutagenesis, a double *imuB*-*imuC* deletion mutant has no further increase in sensitivity or reduction of mutagenesis, strengthening evidence that these genes function in the same pathway in this bacterium [186].

In wild type *C. crescentus*, UV-induced mutagenesis results in a mixture of G:C to A:T transitions, G:C transversions, A:T to G:C or C:G mutations, and tandem substitutions [186]. Under conditions with either *imuB* or *imuC* absent, the mutation spectrum drastically shifts to become dominated by G:C to A:T transitions, with the remainder of mutations being A:T to G:C transitions [186]. The dependence on *imuB* and *imuC* for G:C to C:G transversions, A:T to C:G transitions, and tandem substitutions presents a unique mutation spectrum compared to that of *E. coli umuDC* [56,202,203] or *M. tuberculosis imuA'BC* [165,186]. 

### 7.4. *Streptomyces* and *Streptococcus*

In *Streptomyces coelicolor*, the *dinB2* and *imuC* genes overlap by 4 bp, an organization found in most *Streptomyces* and some other bacteria such as *Sinorhizobium* [204]. In *S. coelicolor*, *imuC* deletion strains have no defect in end patching of telomeres, conjugal transfer, UV survival, or UV‑induced mutagenesis, even though *imuC* is induced by UV exposure and MMC. The authors argue that *imuC* is a rapidly evolving gene and that it may be still developing a new/optimal function [204]. In *Streptococcus uberis*, a mutagenesis cassette has been reported that is induced by UV light and that induces mutagenesis after UV exposure; the genes composing this cassette seem to be present throughout Streptococcacaea [205]. 

### 7.5. Pseudomonas

*P.* *putida* contains an *imuABC* operon [185,206]; it has been shown that during stationary phase mutagenesis *imuC* reduces the frequency of base substitution mutations, whereas *imuB* increases base substitution mutations as well as 1-bp deletions [185]. When *imuC* was deleted, the number of base substitution mutations increased with no change in 1-bp deletions [185]. When *imuB* was deleted, 1-bp frameshifts were decreased with no change seen in base substitution mutations [185]. 

Pol I, an A-family DNA polymerase, can act as a translesion synthesis polymerase [155]. In a *P. putida* Pol I-deficient background, the spontaneous mutation frequency was similar in the presence or absence of *imuC* [206]. However, after UV exposure, A to T and A to G mutations decreased in an *imuC*^−^ strain [206]. Frequency of UV-induced mutations increased two-fold in *imuC*^−^ compared to wild type, but not in a Pol I-deficient background [206]. It was concluded that in an unstressed Pol I deficient background, ImuC does not meaningfully contribute to DNA synthesis, but in the presence of DNA damage ImuC becomes involved in DNA synthesis [206].

In *P. aeruginosa* in response to ciprofloxacin, *imuC* and *dnaE1* are upregulated two- and six-fold, respectively [207]. Also in this species, *imuC* has been shown to be responsible for induced mutagenesis [159]. It must be noted that while *P. aeruginosa imuC* and *P. putida imuC* share 73% identity, they have phenotypically opposite effects [185], where *imuC* (also referred to as *polC* in *P. aeruginosa)* is an anti-mutator in *P. putida* [185,206] and a mutator in *P. aeruginosa* [159]. 

## 8. Prevalence and Diversity of *imuABC* Genes

The *imuABC* genes have only recently garnered attention compared to their relatives, *umuDC* and *dinB*. While the model organism *E. coli* utilizes the *umuDC* family for induced mutagenesis and translesion synthesis, it is now becoming clear that this may be the exception rather than the rule in bacteria [184,208]. The UmuD'_2_C and ImuABC systems are observed to be exclusive; that is, organisms with UmuD and UmuC do not have ImuABC [190]. For example, the shared set of SOS response genes between *E. coli* and *M. tuberculosis* are *lexA*, *recA*, *uvrA*, and a set of inducible polymerase genes: *imuABC* in *M. tuberculosis* and *umuDC* in *E. coli* [184]. The *imuABC* and *umuDC* genes seem to fulfill the same role of induced mutagenesis in the SOS response [198,208].

**Figure 2:**
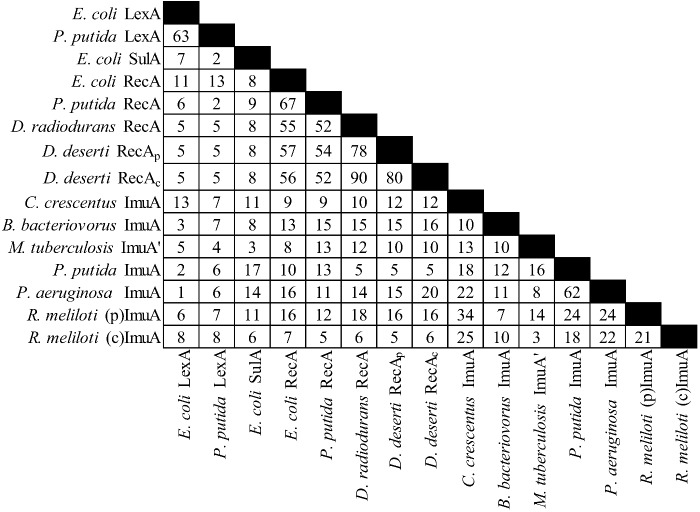
Percent identities by ClustalW2 [213,214] for the ImuA protein and related proteins. “(p)” represents the plasmid gene product, “(c)” represents the chromosomal gene product. The accessions are from Uniprot [215], from left to right: P0A7C2, P0A154, P0AFZ5, P0A7G6, Q07447, P42443, C1D2C5, C1CXY5, C1D2K8, Q9A3J1, Q6MQS4, Q50730, Q88I84, Q9I5Q0, Q92ZJ8, Q92LA5.

**Figure 3:**
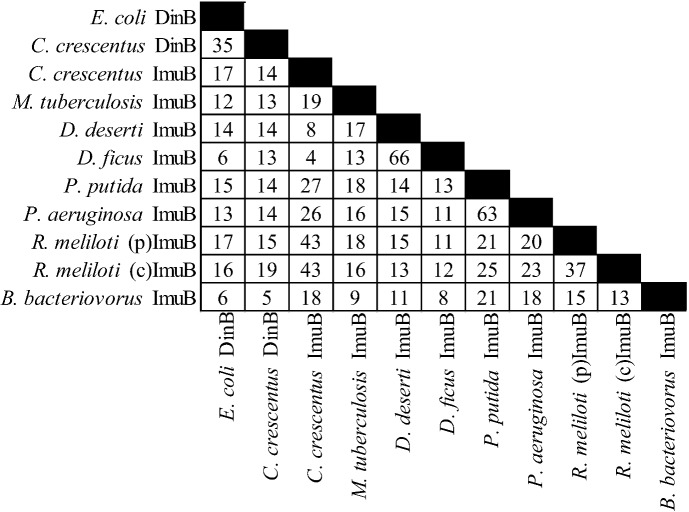
Percent identities by ClustalW2 [213,214] for ImuB protein and related proteins. “(p)” represents the plasmid gene product, “(c)” represents the chromosomal gene product. The accessions are from Uniprot [215] with the exception of *D. ficus* ImuB from RefSeq [216], from left to right: Q47155, Q9A5I1, B8H428, O50419, C1D2K9, ADO33718, Q88I83, Q9I5Q1, Q92ZJ7, Q92JS7, Q6MQS5.

**Figure 4:**
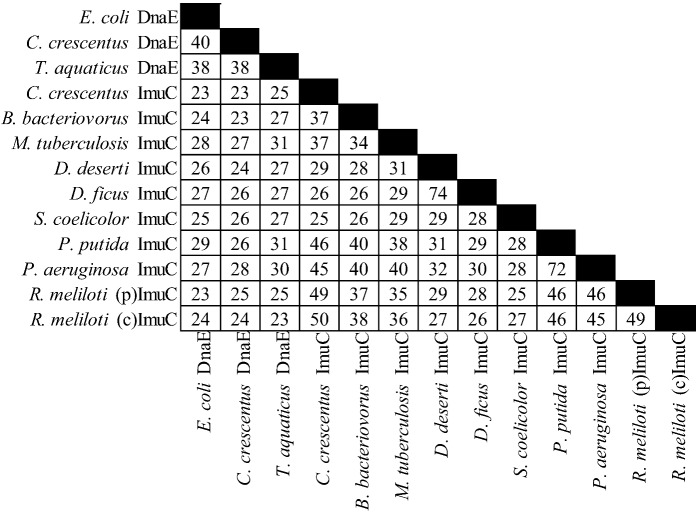
Percent identities by ClustalW2 [213,214] for ImuC proteins and related proteins. “(p)” represents the plasmid gene product, “(c)” represents the chromosomal gene product. The accessions are from Uniprot [215] with the exception of *D. ficus* ImuC from RefSeq [216], from left to right: P10443, B8GWS6, Q9XDH5, B8H427, Q6MQS6, O50399, C1D2L1, ADO33730, Q9S291, Q88I82, Q9I5Q2, Q92ZJ6, Q92LA6.

There is considerable diversity in the gene products of this operon (see Figure 2, Figure 3 and Figure 4). For example *C. crescentus* ImuA and *M. tuberculosis* ImuA' show very little identity. Additionally, various ImuB proteins, such as *S. meliloti* and *P. putida* ImuB (and *D. deserti* ImuY) have few highly conserved residues [190,193]. Even ImuC variants, which are more highly conserved across different organisms, can cause quite different phenotypes. Here *P. aeruginosa* and *P. putida* serve as a prominent example: despite 72% sequence similarity, *P. aeruginosa* ImuC acts as a mutator and *P. putida* ImuC acts as an anti-mutator [159,185,206]. Some species, such as *S. coelicolor*, have an ImuC to which no function has been assigned, although end patching of telomeres, conjugal transfer, UV survival, and UV‑induced mutagenesis have been investigated [204]. 

Transient mutators have the ability to turn on their mutator activity only under stressed conditions, then to turn off their mutator ability to maintain their fitness level once resistance to a selective pressure has been achieved [10,209]. Maintenance of a high mutation frequency under non-stressed conditions would be deleterious to the survival of the organism. SOS-induced mutagenesis is a transient mutator system and in most organisms this response is carried out by the *lexA*-*imuABC* mutagenesis cassette [11,209]. For example, some rifampicin resistant clinical strains of *M. tuberculosis* have high levels of *imuC* expression as a consequence of the same mutation that confers antibiotic resistance [210]. Since these strains have lower fitness in the absence of antibiotic selection, Bergval *et al.* suggest that the reduction in fitness may be due to the inappropriate expression of *imuC*, which is known to have mutator activity in *M. tuberculosis* [210]. The *imuC-*deficient strains of *M. tuberculosis* are less virulent than wild-type strains, and mice infected with these strains experience lower mortality than those infected with the wild type [165]. Elucidation of how hypermutation is associated with infection [211], but not with antibiotic resistance [212] in *P. aeruginosa* may shed more light on the molecular mechanisms of *imuABC*-mediated mutagenesis and its role in bacterial adaptation. 

## 9. Questions and Conclusions

*E. coli* Y-family DNA polymerases are critical in conferring resistance to various DNA damaging agents including UV light and chemical mutagens. The two Y-family polymerases in *E. coli* are capable of bypassing certain lesions and are also involved in the regulation of DNA replication. Y-family polymerases are important in facilitating mutagenesis, contributing to their involvement in antibiotic resistance. The discovery of the *imuABC* mutagenesis cassette indicates another strategy for bacterial mutagenesis and translesion synthesis. Indeed, ImuC facilitates induced mutagenesis and DNA damage tolerance, and possibly provides a missing link between replicative C-family polymerases and the mutagenic Y-family polymerases. The wide phylogenetic and phenotypic diversity of the *imuABC* cassette makes it a prime case study for how mutagenic cassettes appear, evolve, or disappear, and their effects on survival, adaptation, and resistance. 

The discovery of the mutagenesis cassettes that include *imuA*, *imuB*,and *imuC* genes (Table 2) raises a number of questions. One key question is whether *imuC* will demonstrate specificity for certain types of damage, as has been observed for Y-family DNA polymerases. As the number of DNA polymerases and the apparent complexity of DNA damage responses in bacteria continue to increase, a key question is how these polymerases are managed and how access to the replication fork is controlled in response to DNA damage. The possible functions of accessory factors in DNA damage recognition as well as access to the replication fork also remain to be elucidated.
